# Use of the Electronic Nose as a Screening Tool for the Recognition of Durum Wheat Naturally Contaminated by Deoxynivalenol: A Preliminary Approach

**DOI:** 10.3390/s110504899

**Published:** 2011-05-04

**Authors:** Anna Campagnoli, Federica Cheli, Carlo Polidori, Mauro Zaninelli, Oreste Zecca, Giovanni Savoini, Luciano Pinotti, Vittorio Dell’Orto

**Affiliations:** 1 Università Telematica San Raffaele Roma, via di Val Cannuta, 247, 00166, Roma, Italy; E-Mails: carlo.polidori@unisanraffaele.gov.it (C.P.); mauro.zaninelli@unisanraffaele.gov.it (M.Z.); 2 Dipartimento di Scienze e Tecnologie Veterinarie per la Sicurezza Alimentare, Facoltà di Medicina Veterinaria, Università degli Studi di Milano, via Celoria, 10, 20133 Milano, Italy; E-Mails: federica.cheli@unimi.it (F.C.); giovanni.savoini@unimi.it (G.S.); luciano.pinotti@unimi.it (L.P.); vittorio.dellorto@unimi.it (V.D.); 3 Dipartimento di Prevenzione Veterinaria, Azienda Sanitaria Locale di Sondrio, via Stelvio, 35, 23100, Sondrio, Italy; E-Mail: o.zecca@asl.sondrio.it (O.Z.)

**Keywords:** electronic nose, screening methods, durum wheat, deoxynivalenol, PCA, CART

## Abstract

Fungal contamination and the presence of related toxins is a widespread problem. Mycotoxin contamination has prompted many countries to establish appropriate tolerance levels. For instance, with the Commission Regulation (EC) N. 1881/2006, the European Commission fixed the limits for the main mycotoxins (and other contaminants) in food. Although valid analytical methods are being developed for regulatory purposes, a need exists for alternative screening methods that can detect mould and mycotoxin contamination of cereal grains with high sample throughput. In this study, a commercial electronic nose (EN) equipped with metal-oxide-semiconductor (MOS) sensors was used in combination with a trap and the thermal desorption technique, with the adoption of Tenax TA as an adsorbent material to discriminate between durum wheat whole-grain samples naturally contaminated with deoxynivalenol (DON) and non-contaminated samples. Each wheat sample was analysed with the EN at four different desorption temperatures (*i.e.*, 180 °C, 200 °C, 220 °C, and 240 °C) and without a desorption pre-treatment. A 20-sample and a 122-sample dataset were processed by means of principal component analysis (PCA) and classified via classification and regression trees (CART). Results, validated with two different methods, showed that it was possible to classify wheat samples into three clusters based on the DON content proposed by the European legislation: (a) non-contaminated; (b) contaminated below the limit (DON < 1,750 μg/kg); (c) contaminated above the limit (DON > 1,750 μg/kg), with a classification error rate in prediction of 0% (for the 20-sample dataset) and 3.28% (for the 122-sample dataset).

## 1. Introduction

Wheat is a cereal crop that represents one of the most important food and feed commodities. Durum wheat (*Triticum durum* Desf.) products, such as semolina and flour, and wheat by-products derived from the milling process can be characterised by their unique nutritional and functional properties for both human and animal populations. However, durum wheat may also contain detrimental components [1,2], with mould (filamentous fungi) contamination representing one of the most widespread problems. Several issues are associated with grain moulds and their toxic secondary metabolites, known as mycotoxins, including lowered grain quality and, above all, effects on human and animal health [3–5]. In particular, *Fusarium graminearum* and *Fusarium culmorum* are considered the most important deoxynivalenol (DON)-producing species. DON is a mycotoxin belonging to the trichothecenes group, and it represents one of the most frequent mycotoxins in wheat in the more temperate regions of the world, including several European nations [6,7]. DON is chemically described as 12,13-epoxy-3α,7α,15-trihydroxytrichothec-9-en-8-one and has been classified by the International Agency for Research on Cancer [8] in Group 3, *i.e.*, it is not classifiable as to its carcinogenicity for humans. Furthermore, DON represents an important problem in livestock because of its effects that range from feed refusal, vomiting and nausea to immunosuppression and loss of productivity [7,9,10]. The importance and frequency of the occurrence of DON contamination in durum wheat was underscored when, with Commission Regulation (EC) no. 1881/2006 [11], the European Union fixed a specific limit for unprocessed durum wheat among all mycotoxins (coupled with oat and unprocessed maize) only in the case of DON (1,750 μg/kg *vs.* 1,250 μg/kg for all other unprocessed cereals). Because of its high economic and sanitary impact, both the international authorities and the grain industry consider DON contamination as a principal public health concern. At the same time, the monitoring of raw materials destined for industrial milling processes and their derived products represent primary objectives for durum wheat manufacturers, including adequate surveillance and frequent checks [7]. In recent years, a number of cost-effective and fit-for-purpose approaches have been proposed to determine the effectiveness of the safety measures and to achieve logistical and operational goals.

Referring to rapid analytical approaches that provide qualitative or semi-quantitative results, electronic noses (ENs) represent a group of well-known devices for many chemical and microbiological applications, and they have been widely and successfully used in the quality control of food and beverages [12]. An electronic nose (EN) consists of an array of non-specific chemical detectors that interact with different volatile compounds and provide signals that can be utilised effectively as a fingerprint of the volatile molecules rising from the analysed samples. After achieving a fingerprint, identifying and/or quantifying odours by means of a pattern recognition system becomes possible [13]. With regard to the application of ENs in the evaluation of quality changes in grains and the recognition of the presence of fungi and/or mycotoxins, several studies have shown the capability of ENs in discriminating between non-infected samples and samples infected by different species or different strains of toxigenic fungi through the analysis of the produced volatile secondary metabolites [14–19]. In fact, a large group of volatile compounds in cereals has been recognised and are acknowledged to be products of fungal metabolism [20]. The biosynthesis of odourous volatiles from fungal metabolism in cereals is strictly related to environmental conditions of growth. For instance, differences in substrates for metabolic activities are evident in the case of naturally occurring fungal contamination compared with *in vitro* cultures. Despite these aspects, a large number of volatiles can be used as taxonomic markers of mycotoxigenic and non-mycotoxigenic fungi species [15], and a direct relation between volatile compounds and mycotoxin concentration in cereals has been observed [21].

No studies have been previously reported regarding the use of ENs for the classification of cereal samples naturally contaminated by a specific mycotoxin at different levels of contamination. In the present study, an EN constituted by an array of metal oxide semiconductor (MOS) sensors and equipped with a Tenax TA thermal adsorbing/desorbing trap was tested with the aim of investigating its capability as a screening tool to classify durum wheat samples on the basis of their DON contamination level with respect to the legal limit designated by the European legislation for DON content in unprocessed durum wheat (1,750 μg/kg).

## 2. Experimental Section

### 2.1. Samples and Chemical Analyses

A total of over of 250 durum wheat (*T. Durum* Desf.) samples were obtained from lots intended for industrial processing from eight different countries (Australia, Canada, France, Italy, Spain, Syria, Turkey, and the US). The final test samples were collected from incremental portions according to Commission Regulation (EC) no. 401/2006 [22]. Samples were collected throughout 2009 and during the spring of 2010; to prevent the generation of further odours and off-odours, they were stored at −18 °C prior to analyses.

Each test sample was analysed for deoxynivalenol (DON) by high-performance liquid chromatography (HPLC) (limit of detection—LOD—10 μg/kg). High-performance liquid chromatography/mass spectrometry (HPLC/MS) was used for the analysis of diacetoxyscirpenol (DAS), fusarenon (FX), neosolaniol (NEO), nivalenol (NIV), HT-2 toxin (HT-2), T-2 toxin (T-2) (LOD 50 μg/kg), ochratoxin A (OTA) (LOD 0.5 μg/kg) and for aflatoxins B1 (AFB1), B2 (AFB2), G1 (AFG1), and G2 (AFG2, LOD 0.1 μg/kg). Non-contaminated samples, with the exception of DON, and the whole group of samples that displayed negative results for all mycotoxins, presented good aspects and presented no extraneous odours to a sensory human evaluation were selected for the subsequent EN analyses.

### 2.2. Electronic Nose Analysis and Data Acquisition

For comparison purposes, each sample selected was analysed either with one of four different thermal desorption pre-treatments or without thermal desorption. The analyses were performed on a PEN2 model EN operating with an EDU2 enrichment and desorption unit (EDU) from Airsense Analytics GmbH (Schwerin, Germany) and equipped with a HSS 32 headspace autosampler (Perichrom Sarl, Saulx-Les-Chartreux, France). The sensor array consisted of ten metal-oxide-semiconductor (MOS) chemical sensors made of a ceramic substrate heated by a wire resistor and coated by a metal oxide semiconducting film. At the operating conditions (high temperature), interactions between the volatiles from the analyte and the sensor surface induce changes in the conductance of the semiconductor. Thus, the ratio G/G_0_ (where G and G_0_ are the resistance of a sensor in a detecting gas and in clean air, respectively) was recorded by the EN dedicated software. The characteristics of the EN sensor array are listed in Table 1.

For the EN analysis, 3 g of each sample (durum wheat kernels) was placed into airtight 10-mL glass vials sealed with a chlorobutyl/PTFE magnetic cap (Chromacol Ltd., Welwyn Garden City, UK). The headspace of each sample was equilibrated in the shaking incubator oven of the headspace sampler at 27 °C for 5 min to standardize the temperature of all of the samples. The sampling system was either connected to the EDU to achieve a thermal desorption pre-treatment or was connected directly to the EN when samples were analysed directly without using the trapping device. After the pre-treatment (or directly after the equilibration period when the EDU was not used), the sampled gas was suctioned into the EN with a flow of 400 mL/min until each sensor’s response curve showed stabilised conductance (150 s). During this time (measurement time), data from the raw sensor signals for each wheat sample were recorded (with a 1-s interval). For experimental purposes, three aliquots of each sample were singularly analysed, and the mean value of the sensor signals from each aliquot was calculated and recorded as a single odour profile. After each sample analysis, charcoal-filtered air was pumped into the circuit and the sensor chambers for 60 s to clean the system and to achieve the baseline prior to a new injection. All parameters involved in the headspace sampling and analysis were optimised to obtain the best compromise between sensor responses and measurement time.

### 2.3. Thermal Desorption Pre-Treatment

The EDU is a microprocessor-controlled device with the capability of automatically trapping and thermally desorbing the samples. This unit is equipped with the absorbent material Tenax-TA, a porous polymer resin based on 2,6-diphenylene oxide. The breakthrough volume (BV), defined as the amount of carrier gas that causes the analyte to move (or “elute”) from the beginning to the end of the adsorbent bed, is usually adopted to characterize the physical behaviour of the trap and the thermal desorption procedure. The most important parameter that influences BV is the adsorbent bed temperature during trapping and desorption. Thus, the discrimination capability of the EN can be improved by choosing the right sampling and desorption temperature [23]. The setting of the EDU implies a number of steps: (a) sampling: the sampling flow is driven through the tube containing the adsorbent resin; the duration (in the described experiment: 300 s), temperature (27 °C) and flow rate (400 mL/min) need to be chosen; (b) post-sampling: disturbing gaseous compounds and water are removed from the adsorbent; (c) desorption: the trapped compounds are released by heating the adsorbent tube to a defined temperature; (d) injection: the trapped compounds are transferred from the tube into the detector; (e) cleaning: the temperature of the trap is strongly increased according to the given cleaning temperature (280 °C), and a flow of clean gas (charcoal-filtered air) is forced through the system to the waste outlet; (f) cooling: the tube is cooled to reach the starting sampling temperature for treatment of the next sample. In our experiment, four different desorption temperatures (*i.e.*, 180 °C, 200 °C, 220 °C, and 240 °C) were applied to each sample.

Stated the possibility to perform more steps of the analytical procedure simultaneously by the use of the autosampler, the analysis for each sample lasted 860 s when the thermal desorption pre-treatment was applied, whereas without the pre-treatment the total measurement time was 210 s.

### 2.4. Feature Extraction and Data Processing

**Figure 1.**
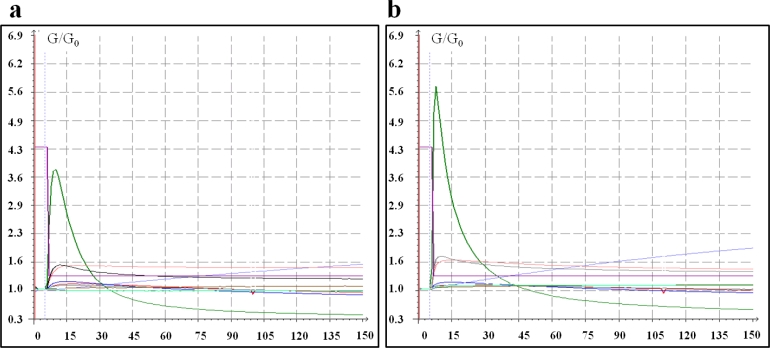
Representative sensor patterns of a negative sample pretreated at two different desorption temperatures. X-axis: Changes of conductance expressed as G/G_0_; Y-axis: Measurement duration (150 s). **(a)** desorption temperature: 180 °C; **(b)** desorption temperature: 200 °C.

Figure 1 represents two examples of the characteristic pattern from the ten sensors of the EN PEN2. Five different datasets were built on the basis of the data from the analyses performed with the four different thermal desorption pre-treatments and without using the trapping device. For each sample, the peak value of the ten raw sensor signals (progressively named “Pic1…Pic10”), the area under the curve (variables named “Area1...Area10”) and the mean value of the last five seconds of measurement (“Last1...Last10”) were recorded. The difference between the peak value of each sensor in comparison to the peak value of the other nine sensors was estimated and recorded (so that a total of 45 variables were recorded): these features were progressively named ”Difp1...Difp45”. The same computation was performed for the area under the curve (variables named “Difa1...Difa45”) and for the mean value of the last five seconds of measurement (“Difs1...Difs45”). In conclusion, each sample was described by 165 variables from EN analysis and one variable from the HPLC analysis of the DON concentration.

On the basis of the level of DON measured by HPLC, each sample was assigned to one of the three classes proposed by the European legislation: a) non-contaminated; b) contaminated below the limit (DON < 1,750 μg/kg) and c) contaminated above the limit (DON > 1,750 μg/kg). For each of the five analytical protocols (without the trapping device and with desorption pre-treatment at 180 °C, 200 °C, 220 °C and 240 °C, respectively) a comparable number of DON-contaminated and non-contaminated samples were selected to optimize the performance of the statistical methods of classification. In particular, the “random under-sampling” approach was adopted, in which class-balance distribution is achieved through random elimination of majority-class examples [24]. According to this approach, the totality of DON-only contaminated samples was grouped with a comparable number of randomly selected non-contaminated samples to build a dataset intended for subsequent statistical analyses.

The classification and regression trees (CART) methodology was then adopted to identify a smaller subset of variables representing those most useful in the discrimination of DON contamination. CART is a non-parametric statistical method that can be used for the classification, regression or selection of features for multivariate data. CART is based on the recursive data-partitioning algorithm, a step-by-step process by which a *decision tree* is constructed by the splitting or non-splitting of each node on the tree into two *daughter nodes*. At the end of the tree, nodes that do not split are called *terminal nodes* and are assigned to a class label. The most interesting feature of these trees is that, because the algorithm consists of a sequence of hierarchical Boolean questions (e.g., is X_i_ < θ_j_? where θ_j_ is a threshold value), each of which depends upon the answers to the previous ones, at the end of the process, it is trivial to highlight which variables of the given input set X_1_, X_2_,…, X_r_ are explanatory for classification purposes. Consequently, a subset of the most significant variables, which can be used as new input data for further linear or non-linear classification or regression methods, can be selected [25].

For each of the five datasets, the subset of variables selected from the 165 original variables via the CART method was subsequently adopted as input data for the matrix-correlation principal component analysis (PCA). This technique transforms the entire set of *n*-correlated variables to *n*-uncorrelated linear functions of the original measurements. The first principal component is the linear combination of all variables showing the maximum variation among the samples. The second, third and further components are similarly linear combinations representing the next-largest variations, irrespective of those represented by the previous combination. In the literature, orthogonal transformations such as PCA have been utilized to mitigate possible negative effects of a feature-selection process (as in the case of CART model), such as the correlation among variables of datasets [26].

Afterwards, to forecast the sample’s class for each record of the five datasets, PCA scores were used as predictors in a new CART application, which was selected from three multivariate discriminant techniques (linear discriminant analysis (LDA), K-nearest neighbours (K-NN) and CART), on the basis of the “cross-validated error-rate” (CVER) and “cross-validated risk” (CVR). Results were validated by the “leave-one-out” method.

Classification performances for each of the five analytical protocols were calculated and the best two were selected. Subsequently, for each one of the two identified best-performing protocols, a new “enlarged” dataset was built that contained the PCA scores of all data from DON-contaminated samples together with the data from the entire group of non-contaminated samples. A ten-partition “K-fold cross-validation” method was applied to the two enlarged datasets with the aim of implementing a more appropriate and strict validation of the classifier [27]. In K-fold cross-validation, the dataset is randomly divided into K subsets, and the holdout method is repeated K times. Each time, one of the K subsets is used as the test set and the other K-1 subsets grouped together constitute the training set. Then, the average error across all K trials is computed. Note that in the case of leave-one-out cross validation, K is equal to the number N of data points in the set and the resulting auxiliary trees would be almost identical to the tree constructed from the full dataset; consequently, nothing would be gained from the procedure [28]. Statistical analyses were implemented using MATLAB Statistics Toolbox (R2010a, The Mathworks, Inc., Natck, MA, USA).

## 3. Results

According to the aim of this study, the results of the multiple mycotoxins contamination analyses (quantification of deoxynivalenol (DON), diacetoxyscirpenol (DAS), fusarenon (FX), neosolaniol (NEO), nivalenol (NIV), ochratoxin A (OTA), HT-2 toxin (HT-2), T-2 toxin (T-2) and aflatoxins (AFB1, AFB2, AFG1, AFG2)) were adopted to select samples characterised only by DON contamination. From the whole group of samples analysed, nine displayed negative results towards all mycotoxins, with the exception of DON, whereas 113 results indicated contamination levels under the LOD for all mycotoxins and, lacking further extraneous odours at human olfactory perception, these samples were labelled as non-contaminated. Six of the nine DON-only naturally contaminated samples showed a mycotoxin level below (500 < DON < 1,500 μg/kg) the limit of 1,750 μg/kg fixed by the European Commission for unprocessed durum wheat (Commission Regulation (EC) no. 1881/2006), whereas three of these samples were characterised by a contamination level above the legal limit (1,800 < DON < 2,500 μg/kg). The nine DON-only contaminated samples were selected for further EN analysis, together with a comparable number (n = 11) of randomly selected, non-contaminated samples. Thus, a total of 20 samples were used to verify the capability of the EN in classifying wheat samples into three clusters based on the DON contamination. Features of the DON-contaminated samples for EN evaluation are summarised in Table 2.

Due to the high number of independent variables (165 descriptors coming from EN sensors’ responses) describing the 20 samples, the first step for the EN data evaluation was to reduce the size of the datasets. Data reduction was implemented by applying the CART algorithm for variable selection. CART is robust to the presence of outliers, and it selects variables that have mutual relations in data classification [29]. In doing so, CART provides a subset of variables directly towards achieving the separation of *a priori* defined classes [27]. Therefore, with the emphasis on obtaining a small subset, the variables selected with the CART algorithm can be used as input data for further linear or non-linear classifications or regression methods [30]. The adoption of the CART algorithm for data reduction in the described experiment is justified by the fact that, despite the pre-selection of DON-only naturally contaminated and non-contaminated samples, it was not theoretically possible to exclude the influence on the EN response to unknown disturbing odour sources due to sources other than mould infections (e.g., volatiles developed during storage, delivery and transport, harvesting, *etc.*). Data variance can be strongly influenced by these causes; thus, a method oriented towards *a priori* defined classes was considered appropriate to point out information regarding DON content.

By applying the CART model to each of the five datasets (four from analyses performed with the thermal desorption pre-treatment and one without the trapping device included), at least five variables were selected so that a massive data reduction was achieved. Results are summarised in Table 3.

**Figure 2.**
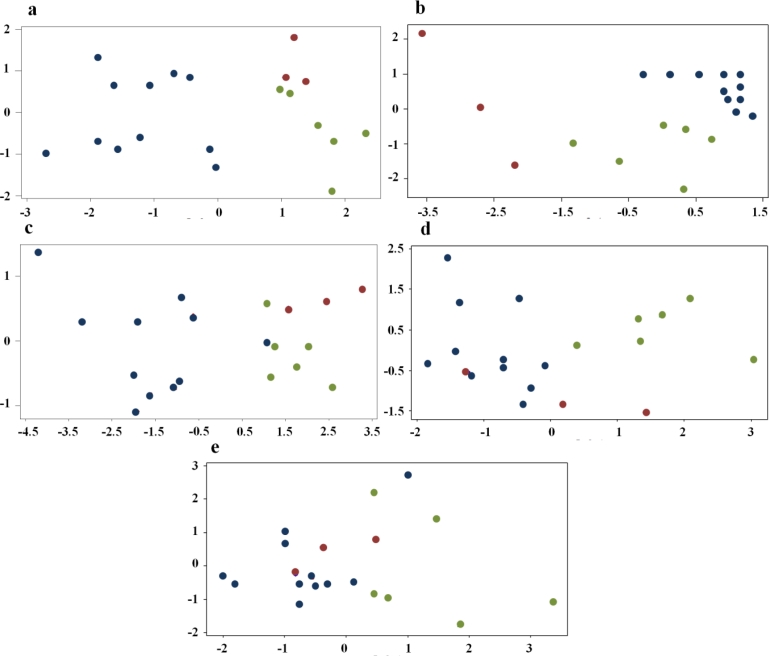
PCA score plots of first (X-axis) and second (Y-axis) principal components of the five analytical protocols’ reduced data. Blue dots: non-contaminated samples; Green dots: contamination level below the legal limit; Red dots: contamination level above the legal limit. **(a)** protocol without the trapping device; **(b)** protocol with desorption at 180 °C; **(c)** protocol with desorption at 200 °C; **(d)** protocol with desorption at 220 °C; **(e)** protocol with desorption at 240 °C.

The five “reduced” datasets were then explored and graphically expressed by the correlation-matrix PCA. The corresponding score plot of the two principal components are presented in Figure 2 for each of the five datasets.

As highlighted by Figure 2, a clear separation among the three classes is evident in the case of the protocol without pre-treatment (the model was able to explain 82.7% of total data variance by the first and second principal components, 57.8% and 24.9%, respectively) and in the case of the desorbing pre-treatment at 180 °C (79.8% of total data variance explained by the first two PCs, 49.7% and 30.1%, respectively). A slightly good separation between the contaminated and non-contaminated samples is observable in the datasets from desorbing pre-treatments at 200 °C (95.6% of total data variance explained by the first two PCs, 86.8% and 8.9%, respectively) and 220 °C (97.1% of total data variance explained by the first two PCs, 63.7% and 33.3%, respectively). The desorbing pre-treatments at 240 °C do not account for a clear class separation on the basis of DON contamination (74.1% of total data variance explained by the first two PCs, 40.6% and 33.4%, respectively).

**Figure 3.**
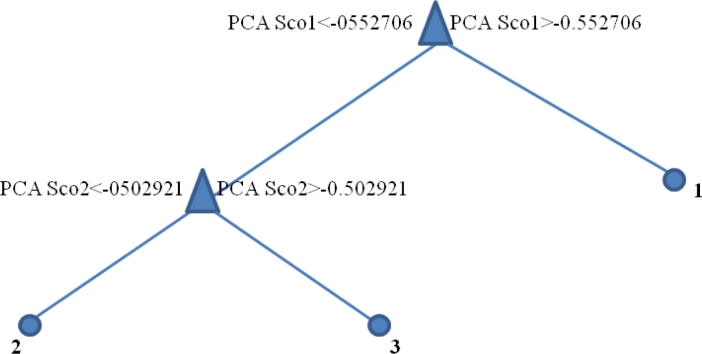
Classification tree of the dataset obtained without thermal desorption. Results from other analytical protocols (thermal desorption pre-treatment at four different temperatures) were similar and are omitted for clarity.

The principal component scores were then recorded and utilized for the final sample classifications on the basis of DON contamination. At this stage, after a selection among three different classification algorithms (LDA, K-NN and CART), CART methodology was selected and adopted for sample classifications by means of PCA data. The CART results showed that, for all of the five reduced datasets, the first and the second PCs were the explanatory variables selected to build the classification tree, except in the case of the analytical desorption protocol at 240 °C, in which only one node was constituted by selecting the first component as the explanatory variable. Figure 3 represents an example of a classification tree from CART for the dataset obtained without thermal desorption: the PC1 score (first node) identified the difference between non-contaminated samples (group 1) and contaminated samples, whereas the PC2 score (second node) split the samples into two groups: below the legal limit (group 2) and above the legal limit (group 3).

The best results were achieved by the protocol in which no thermal desorption pre-treatment was adopted. No errors in class estimations were committed in fitting or in prediction. Good results were also observed from the protocol with a desorption pre-treatment at 180 °C (5% of misclassified samples, both in fitting and prediction). Only one highly contaminated sample was misclassified as contaminated below the legal limit both in fitting and in prediction. Results from the desorption pre-treatment at 200 °C and 220 °C, where three and four samples were misclassified in the prediction, respectively, can be considered similar. The same two protocols give two and one error, respectively, in fitting (10% and 15% of misclassified samples in fitting and in prediction, respectively, at 200 °C; 5% and 20% of misclassified samples in fitting and in prediction, respectively, at 220 °C). As observed in the PCA score plots, in the case of the first two previously described analytical protocols (no thermal desorption and desorption at 180 °C), all three clusters, which are based on DON contamination levels, were accounted by each model. For two other protocols (200 °C and 220 °C thermal desorption protocols) the three clusters were accounted but misclassification increased. A true and clear distinction among the three classes based on DON contamination was not observable in the case of the 240 °C thermal desorption protocol, in which only two clusters were accounted (non-contaminated and contaminated samples) and 20% and 25% of samples were misclassified in fitting and in prediction, respectively. On the basis of these results, the simpler analytical protocol (without the trapping device) could be considered the most efficient (no errors in fitting, nor in prediction), whereas the second-best-performing protocol is that characterized by the pre-treatment at 180 °C, in which one error in fitting and one in prediction were accounted, respectively.

**Figure 4.**
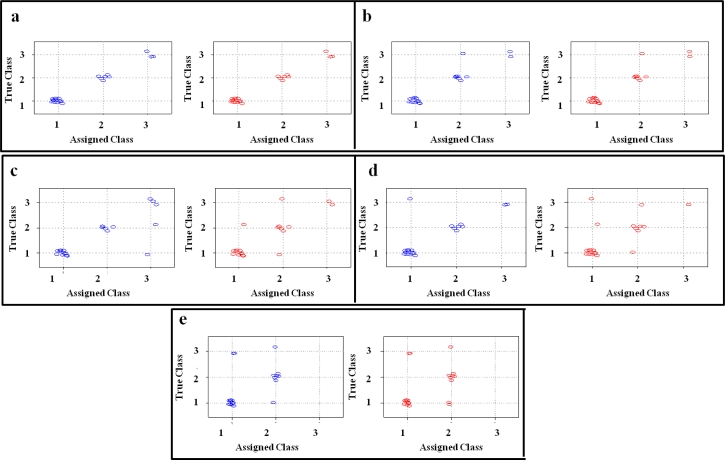
CART-model performance plots applied to each analytical protocol. Blue dots: samples classification in fitting; Red dots: samples classification in prediction by leave-one-out cross-validation). **(a)** protocol without the trapping device; **(b)** protocol with desorption at 180 °C; **(c)** protocol with desorption at 200 °C; **(d)** protocol with desorption at 220 °C; **(e)** protocol with desorption at 240 °C.

Graphical representations of CART classification performances were also possible. Performance plots for each analytical protocol are presented in Figure 4. Classes assigned by the model are represented on the X-axis, whereas the true classes are represented on the Y-axis. The group of non-contaminated samples is on the left-bottom part of each plot; samples below the legal limit are on the central part of the plots; and samples above the legal limit are on upper-right part of the plots. In Table 4, the performances of the models in both fitting and prediction are summarised.

**Figure 5.**
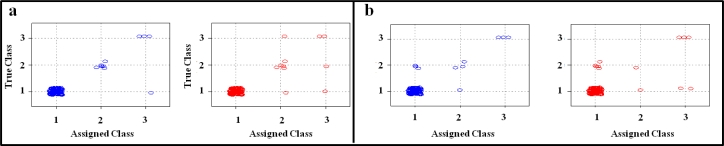
Classification plots from CART model applied to the two “enlarged” datasets. Blue dots: samples classification in fitting; Red dots: samples classification in prediction (using 10-fold cross-validation). **(a)** protocol without the trapping device; **(b)** protocol with desorption pre-treatment at 180 °C.

Finally, the selected variables referred to the two best-performing analytical protocols (the protocol without the use of the trapping device and the protocol with a desorption temperature of 180 °C) were considered to build two new “enlarged” datasets (with nine DON-contaminated and 113 non-contaminated samples) that were submitted once again to CART classification using a 10-fold cross-validation method. As expected, classification performances were lower than in the case of the 20-sample datasets tested with the leave-one-out method; however, as in the case of the 20-sample dataset, the best results were achieved by the protocol with no thermal desorption pre-treatment. Results from validation applied to the protocol without the desorption pre-treatment showed that only one non-contaminated sample was misclassified as highly contaminated in fitting, whereas in prediction, two non-contaminated samples were classified as DON-contaminated; one highly contaminated sample was misclassified as contaminated below the legal limit and one sample below the legal limit was misclassified as highly contaminated (fitting error rate and risk: 0.82%; prediction error rate and risk: 3.28%). Results from the application of the desorption pre-treatment at 180 °C in fitting showed that three positive samples below the limit were misclassified as negative, whereas a non-contaminated sample was classified as contaminated below the legal limit (error rate and risk: 3.28%). More forecasting errors were evident in prediction (error rate and risk: 6.56%). Differences between the two protocols were evident. One (without the desorption pre-treatment) and four (at 180 °C desorption temperature) samples in fitting and four (without the desorption pre-treatment) and eight (at 180 °C desorption temperature) samples in prediction were misclassified, respectively. Figure 5 and Table 5 show CART performance plots, both in fitting and in prediction, using 10-fold cross-validation for the protocol without the trapping device and for the protocol with desorption pre-treatment at 180 °C, respectively.

## 4. Discussion

In many cases, currently available analytical techniques that are suited for the detection and quantification of volatile compounds from fungi metabolism have demonstrated their capacity to detect and quantify volatiles at a very early stage of fungal development and under the human olfactory perception threshold. Olsson *et al.* identified and quantified several volatiles from mycotoxigenic fungi metabolism in DON-contaminated barley grain [21]. They found a positive correlation between the concentration of five compounds (pentane, methylpyrazine, 3-pentanone, 3-octene-2-ol and isooctylacetate) and the level of DON contamination, whereas six molecules (ethylhexanol, pentadecane, toluene, 1-octanol, 1-nonanol, and 1-heptanol) showed a negative correlation with the mycotoxin. The same paper described similar results in the case of ochratoxin A [21]. A number of analytical techniques involve adsorbing/desorbing techniques as pre-treatments of gaseous analytes, *i.e.*, solid-phase micro-extraction (SPME) or thermal approaches based on porous polymer resins [7,12,31]. In the present study, different thermal desorption protocols based on Tenax TA as an adsorbent material were utilised according to the breakthrough volumes (BVs) of the group of compounds presumably associated with DON mycotoxin contamination. The use of four different desorption temperatures allowed coverage of the entire space of BVs for all possible DON markers. Despite these attempts, the best results in classifying samples on the basis of DON contamination were achieved by an EN analytical protocol without the thermal desorption pre-treatment. In this case, with a 20-sample, balanced-classes (nine DON-contaminated and 11 non-contaminated) dataset, no errors in fitting or in prediction were accounted by the classification model validated by the leave-one-out method. The performance of the simplest analytical protocol (without desorption pre-treatment) resulted in the best results after further validation of the classifier applied to the “enlarged” 122-sample dataset. In the latter case, a distribution among classes of nine DON-contaminated and 113 non-contaminated samples more faithfully reproduced a real-life situation characterised by unbalanced classes; in this case, a more appropriate 10-fold cross-validation was adopted. As expected, classifying performance was lower than in the 20-sample dataset case, and four errors were computed in prediction; however, none of the contaminated samples were misclassified as non-contaminated, avoiding the worst eventuality under *in-field* conditions. The second-best classifying performance was achieved by data from the analytical protocol with a thermal desorption pre-treatment at 180 °C, but considering the disadvantages due to a longer duration of the analysis (860 s with pre-treatment *vs.* 210 s without pre-treatment), this choice does not seem practical. Furthermore, classification performance appeared to worsen as the desorbing temperature increased. CART methodology was applied for both variable selection (first step) and sample classification (second step). In summary, the instrument showed a satisfactory capability in classifying between non-contaminated and contaminated samples below and above 1,750 μg/kg, the legal limit of DON concentration in durum wheat established by the European Union.

Results obtained by the EN equipped with MOS sensors may represent an interesting preliminary outcome that suggests several possible applications of this instrument as a mycotoxin screening tool. As a general rule, most rapid methods that provide qualitative or semi-quantitative results are recommended in sample screening. An analytical method is usually referred to as “rapid” when it requires, at most, a few minutes to obtain a result [32]. In the described experiment, the best results were achieved by an analytical protocol that required less than four minutes for each analysis. However, for mycotoxin screening, measurement speed is not the only factor to be considered. Other parameters are also fundamental, such as reliability, non-destructivity, cost of analysis, the possibility of *in-field* use and the skill level required to perform the assay [33]. In the above-described experiment, analysis was totally non-destructive, the instrument accounts for a very low exercise cost for single analyses (*i.e.*, no chemicals are required), it is capable of working well under *in-field* conditions, and after an adequate training period, it can be utilised in an almost completely automated way. Furthermore, the EN can face more than one analytical problem with minimal changes to its settings.

## 5. Conclusions

Results obtained by the described experiment indicate that the EN equipped with MOS sensors allows the classification of naturally contaminated samples on the basis of DON content. Four analytical protocols using thermal desorption by Tenax-TA as the adsorbent material were applied at different working temperatures (*i.e.*, 180 °C, 200 °C, 220 °C, and 240 °C, respectively). One analytical protocol without the use of a desorption pre-treatment was also tested. Good results were achieved at a desorption temperature of 180 °C, but the best results were obtained with the simplest analytical protocol, in which no thermal desorption procedure was adopted and the duration of the analysis was short (210 s). The approach described here allowed us to classify analysed samples into three classes on the basis of the European Union limits for DON in unprocessed durum wheat: (a) non-contaminated; (b) contaminated below the limit (DON < 1,750 μg/kg); (c) contaminated above the limit (DON > 1,750 μg/kg); with a prediction error rate of 0% when a 20-sample dataset was validated by the leave-one-out method. Reasonable results were also achieved with the 122-sample dataset, for which a k-fold cross-validation was applied; this method showed a prediction error rate of 3.28%.

Even considering that the analytical method tested with this experiment was essentially in the initial phase of validation, and taking into account the peculiar conditions under which this test was set up (only DON as contaminant, good quality of samples with absence of extraneous odours), the simple analytical protocol described here, which combined the application of the CART model and PCA for the selection of variables and the classification of samples, seems to provide encouraging results. Thus, we hope that the method described here can be tested on more complex analytical matrices, particularly samples contaminated with multiple mycotoxins, to verify the EN capability in rapid selection, on a “yes or no” criterion basis, of samples that undergo more expensive and time-consuming quantitative analyses.

## Figures and Tables

**Table 1.** MOS Sensor Array of PEN2 and their main application.

**No. in array**	**Sensor name**	**Description**	**Reference**
1	W1A-aromatic	Aromatic compound	Toluene, 10 mg/kg
2	W5B-broadrange	Broad range sensitivity reacts to nitrogen oxides and ozone very sensitive with negative signal	NO_2_, 10 mg/kg
3	W3A-aromatic	Ammonia, used as sensor for aromatic compounds	Benzene, 10 mg/kg
4	W6B-hydrogen	Mainly hydrogen, selectively (breath gases)	H_2_, 100 mg/kg
5	W5A-arom-aliph	Alkanes, aromatic compounds, less polar compounds	Propane, 1 mg/kg
6	W1B-broad-methane	Sensitive to methane (environment) ca. 10 mg/kg Broad range, similar to No. 8	CH_4_, 100 mg/kg
7	W1C-sulphur-organic	Reacts on sulphur compounds H_2_S 0.1 mg/kg. Otherwise sensitive to many terpenes and sulphur organic compounds, which are important for smell, limonene, pyrazine.	H_2_S, 1 mg/kg
8	W2B-broad-alcohol	Detects alcohols, partially aromatic compounds, broad range	CO, 100 mg/kg
9	W2C-sulphur-chlor	Aromatics compounds, sulphur organic compounds	H_2_S, 1 mg/kg
10	W3B-methane-aliph	Reacts on high concentrations >100 mg/kg, sometimes very selective (methane)	CH_4_, 10 mg/kg

**Table 2.** DON contamination in wheat samples. Class Assignment **(b)** samples characterised by a DON level below the limit fixed by the European Commission for unprocessed durum wheat; Class Assignment **(c)** samples characterised by a mycotoxin level above the legal limit.

**Sample**	**DON (μg/kg)**	**Class Assignment**
1	400	b
2	500	b
3	500	b
4	900	b
5	1,000	b
6	1,500	b
7	1,800	c
8	2,000	c
9	2,500	c

**Table 3.** Variables selected by the CART algorithm.

**Analytical protocol**	**1° variable selected**	**2° variable selected**	**3° variable selected**	**4° variable selected**	**5° variable selected**
No thermal desorption pre-treatment	difa13	difa15	difa37	difp29	
Desorption temperature 180 °C	difl14	difa26	difa43	difp2	
Desorption temperature 200 °C	difl40	difl43	difl44	difa15	difa36
Desorption temperature 220 °C	difl5	difp1	difl13		
Desorption temperature 240 °C	area10	difa32	difp45	difl17	

**Table 4.** CART classification performances. Class **a)**, non-contaminated samples; Class **b)**, samples contaminated below the legal limit; Class **c)**, samples contaminated above the legal limit.

				**Misclassification matrix (Samples fitted assignment)**			**Cross-validated misclassification matrix (Samples predicted assignment)**		
**Analytical protocol**	**True class**	**Total true samples**		**Assigned class**			**Assigned class**		
				**a**	**b**	**c**	**a**	**b**	**c**
**No thermal desorption pre-treatment**	**a**	11		11	0	0	11	0	0
Error rate: 0.000			**rate**	1.000	0.000	0.000	1.000	0.000	0.000
Risk: 0.000	**b**	6		0	6	0	0	6	0
Cross-validated Error Rate: 0.000			**rate**	0.000	1.000	0.000	0.000	1.000	0.000
Cross-validated Risk: 0.000	**c**	3		0	0	3	0	0	3
			**rate**	0.000	0.000	1.000	0.000	0.000	1.000
**Desorption temperature 180 °C**	**a**	11		11	0	0	11	0	0
Error rate: 0.050			**rate**	1.000	0.000	0.000	1.000	0.000	0.000
Risk: 0.050	**b**	6		0	6	0	0	6	0
Cross-validated Error Rate: 0.050			**rate**	0.000	1.000	0.000	0.000	1.000	0.000
Cross-validated Risk: 0.050	**c**	3		0	1	2	0	1	2
			**rate**	0	0.333	0.667	0	0.333	0.667
**Desorption temperature 200 °C**	**a**	11		10	0	1	10	1	0
Error rate: 0.100			**rate**	0.909	0.000	0.901	0.909	0.091	0.000
Risk: 0.100	**b**	6		0	5	1	1	5	0
Cross-validated Error Rate: 0.150			**rate**	0.000	0.833	0.167	0.167	0.833	0.000
Cross-validated Risk: 0.150	**c**	3		0	0	3	0	1	2
			**rate**	0.000	0.000	1.000	0.000	0.333	0.667
**Desorption temperature 220 °C**	**a**	11		11	0	0	10	1	0
Error rate: 0.050			**rate**	1.000	0.000	0.000	1.909	0.091	0.000
Risk: 0.050	**b**	6		0	6	0	1	5	0
Cross-validated Error Rate: 0.200			**rate**	0.000	1.000	0.000	0.167	0.833	0.000
Cross-validated Risk: 0.200	**c**	3		1	0	2	1	1	1
			**rate**	0.333	0.000	0.667	0.333	0.333	0.333
**Desorption temperature 240 °C**	**a**	11		10	1	0	9	2	0
Error rate: 0.200			**rate**	0.909	0.901	0.000	0.818	0.182	0.000
Risk: 0.200	**b**	6		0	6	0	0	6	0
Cross-validated Error Rate: 0.250			**rate**	0.000	1.000	0.000	0.000	1.000	0.000
Cross-validated Risk: 0.250	**c**	3		2	1	0	2	1	0
			**rate**	0.667	0.333	0.000	0.667	0.333	0.000

**Table 5.** Performances of classification by CART for the two enlarged datasets. Class **a)**, samples non-contaminated; Class **b)**, samples below the legal limit; Class **c)**, samples above the legal limit.

				**Misclassification matrix Samples fitted assignment)**			**Cross-validated misclassification matrix (Samples predicted assignment)**		
**Analytical protocol**	**True class**	**Total true samples**		**Assigned class**			**Assigned class**		
				**a**	**b**	**c**	**a**	**b**	**c**
**No thermal desorption pre-treatment**	**a**	113		112	0	1	111	1	1
Error rate: 0.0082			**rate**	0.991	0.000	0.009	0.982	0.009	0.009
Risk: 0.0082	**b**	6		0	6	0	0	5	1
Cross-validated Error Rate: 0.0328			**rate**	0.000	1.000	0.000	0.000	0.833	0.167
Cross-validated Risk: 0.0328	**c**	3		0	0	3	0	1	2
			**rate**	0.000	0.000	1.000	0.000	0.333	0.667
**Desorption temperature 180 °C**	**a**	113		112	1	0	110	1	2
Error rate: 0.0328			**rate**	0.991	0.009	0.000	0.973	0.009	0.018
Risk: 0.0328	**b**	6		3	3	0	5	1	0
Cross-validated Error Rate: 0.0656			**rate**	0.500	0.500	0.000	0.833	0.167	0.000
Cross-validated Risk: 0.0656	**c**	3		0	0	3	0	0	3
			**rate**	0.000	0.000	1.000	0.000	0.000	1.000
